# Carbon dots labeled *Lactiplantibacillus plantarum:* a fluorescent multifunctional biocarrier for anticancer drug delivery

**DOI:** 10.3389/fbioe.2023.1166094

**Published:** 2023-05-25

**Authors:** Noor A. Abdullah, Hoda E. Mahmoud, Nefertiti A. El-Nikhely, Ahmed A. Hussein, Labiba K. El-Khordagui

**Affiliations:** ^1^ Department of Biotechnology, Institute of Graduate Studies and Research, Alexandria University, Alexandria, Egypt; ^2^ Department of Pharmaceutics, Faculty of Pharmacy, Alexandria University, Alexandria, Egypt

**Keywords:** bioimaging, probiotics, prodigiosin, heat-inactivated *L. plantarum*, Caco-2 cells, MTT assay, apoptosis, molecular docking

## Abstract

A carbon dots (CDs)-biolabeled heat-inactivated *Lactiplantibacillus plantarum* (HILP) hybrid was investigated as a multifunctional probiotic drug carrier with bioimaging properties using prodigiosin (PG) as anticancer agent. HILP, CDs and PG were prepared and characterized using standard methods. CDs-labeled HILP (CDs/HILP) and PG loaded CDs/HILP were characterized by transmission electron microscopy (TEM), laser scanning confocal microscopy (LSCM) and for entrapment efficiency (EE%) of CDs and PG, respectively. PG-CDs/HILP was examined for stability and PG release. the anticancer activity of PG-CDs/HILP was assessed using different methods. CDs imparted green fluorescence to HILP cells and induced their aggregation. HILP internalized CDs via membrane proteins, forming a biostructure with retained fluorescence in PBS for 3 months at 4°C. Loading PG into CDs/HILP generated a stable green/red bicolor fluorescent combination permitting tracking of both drug carrier and cargo. Cytotoxicity assay using Caco-2 and A549 cells revealed enhanced PG activity by CDs/HILP. LCSM imaging of PG-CDs/HILP-treated Caco-2 cells demonstrated improved cytoplasmic and nuclear distribution of PG and nuclear delivery of CDs. CDs/HILP promoted PG-induced late apoptosis of Caco-2 cells and reduced their migratory ability as affirmed by flow cytometry and scratch assay, respectively. Molecular docking indicated PG interaction with mitogenic molecules involved in cell proliferation and growth regulation. Thus, CDs/HILP offers great promise as an innovative multifunctional nanobiotechnological biocarrier for anticancer drug delivery. This hybrid delivery vehicle merges the physiological activity, cytocompatibility, biotargetability and sustainability of probiotics and the bioimaging and therapeutic potential of CDs.

## 1 Introduction

Probiotics, nonpathogenic bacteria that inhabit the gastrointestinal tract (GIT), are currently under active investigation in biotherapy owing to their recognized safety, multiple health benefits and physiological functions (Gomaa, 2020; Yu and Tian, 2020). GIT probiotics naturally regulate the growth of other microorganisms, intestinal permeability, digestion, and metabolism. They are also connected to human physiology through crosstalk with different body sites and the immune system, driving changes in the response to drug therapies as well as the prevention, onset, and progression of several diseases including cancer (Javid et al., 2023; Nami et al., 2023) and non-cancer conditions (Un-Nisa et al., 2023; Xia et al., 2023).

Such characteristics led to the wide use of probiotics as bioactive agents in nutritional and pharmaceutical products (Lee et al., 2018) as well as biocarriers for the administration of drugs and vaccines (Kang et al., 2022). Gram-positive *Lactobacillus* and *Bifidobacterium* rods, *Saccharomyces cerevisiae* var. *boulardii* in addition to the non-pathogenic *Escherichia coli* Nissle 1917 are the most widely investigated probiotics in this respect (Chen et al., 2023). As drug biocarriers, probiotics offer advantages compared with nano-probiotic bacteria and conventional pharmaceutical drug delivery systems. While probiotics retain the bioactivity and biotargeting functionality of nonprobiotic bacteria, their non-pathogenicity reduces the risk of infection and allows for safer use, particularly in vulnerable cases. In comparison with biomaterial-based drug carriers, probiotics are characterized by sustainability, scalability, relatively low cost, inherent biotargeting ability with possible spacio-temporal control over drug release (Xie et al., 2021). Moreover, a properly selected probiotic may act as therapeutic adjuvant to improve the efficacy and safety of the probiotic-cargo combination and restore homeostasis within a perturbed microbial community (Javid et al., 2023).

The versatile probiotic-based biocarrier platform includes micro-sized structures such as live probiotics, inactivated and ghost cells in addition to spores (Han et al., 2022) as well as nanosized probiotic-derived structures. These include extracellular or outer membrane vesicles (Dean et al., 2020) and minicells (Nguyen et al., 2017) as more specific and safer alternatives to bacteria. Probiotic cells have been greatly enhanced by synthetic biology and nanotechnology tools. For instance, probiotic ghosts, non-viable cells that eliminate safety and shelf-life limitations associated with probiotics, proved suitable as biocarriers for vaccines and drugs (Hou et al., 2018; Saleh et al., 2022). Yeast cells, either living, dead or ghost have also been used for the delivery of various bioactive agents (Coradello and Tirelli, 2021). Moreover, genetically modified probiotics may act as biocarriers for in situ-expressed therapeutic proteins and peptides (Charbonneau et al., 2020; Nakagawa et al., 2022) as well as probiotic-synthesized inorganic nanomaterials (Mohammed et al., 2023; Rabiee et al., 2023). Further advancement of probiotics as drug delivery biocarriers may be greatly enhanced by bioimaging strategies. These would allow visualization of the movement of the probiotic/cargo combination *in vivo* and their potential interactions with target cells *in vitro*.

In this context, we present herein an innovative fluorescent *L. plantarum* (LP) as trackable hybrid drug biocarrier for potential colorectal cancer (CRC) therapy using prodigiosin (PG) as anticancer agent. LP, formerly *Lactobacillus plantarum*, was selected as biocarrier because of its recognized safety, tolerance toward GIT environment as well as adhesion and cytotoxicity to CRC cells (Ahmad et al., 2018; Zhao et al., 2022). HILP was used for further improved probiotic safety and keeping properties, given that heat inactivation may not significantly affect the surface characteristics of *Lactobacillus* spp. and their bioactivity (Ciandrini et al., 2017). Although organic dyes (Zhao et al., 2022), metal-based quantum dots (Butrimienė et al., 2022) and fluorescent proteins produced by genetically engineered probiotic cells (Han et al., 2015) were commonly documented as probiotic labeling agents, fluorescent carbon dots (CDs) were used in the present study because of their sustainability, cytocompatibility, non-toxicity, and high fluorescence stability (Nandi et al., 2015; Unnikrishnan et al., 2020). CDs are a relatively new type of fluorescent nanomaterials with excellent photoluminescence (Jing et al., 2023). Indeed, CDs labeling is a versatile, simple, and inexpensive technique of value in selective biolabeling of bacteria, tumor cells, proteins, and subcellular organelles for a wide range of bioimaging and theranostic purposes (Hallaji et al., 2022; Phan and Cho, 2022; Fu et al., 2023). Moreover, CDs were widely investigated as fluorescent carriers for targeted drug delivery (Boakye-Yiadom et al., 2019). However, to the best of our knowledge, CDs-labeled probiotics as biocarriers for drug delivery applications have not been documented to date.

Prodigiosin (PG) used as anticancer agent for the functionalization of the CDS/HILP hybrid is a red pigment having a tripyrrole ring core structure produced by *Serratia marcescens.* PG shows a wide spectrum of biological activities including distinct anticancer activity against different cancers without harmful effects on normal cells (Islan et al., 2022). This rendered PG a promising candidate for drug development and the design of drug delivery systems of the conventional polymer type (Araujo et al., 2022) and bacteria-based types (Saleh et al., 2022) for bioavailability and activity enhancement. Loading of red fluorescent PG into CDs/HILP would produce a bicolor fluorescent structure for distinct bioimaging of the drug carrier and cargo.

The study aimed at the development of a carbon dots-labeled fluorescent probiotic hybrid and its characterization as a trackable biocarrier for drug delivery applications using prodigiosin as anti-colorectal cancer agent. Emphasis was placed on the effects of CDs on the morphological characteristics of HILP cells as well as the uptake and retention of CDs by HILP and preservation of the hybrid fluorescence in aqueous media. Moreover, contribution of the bioactivity and bioimaging ability of CDs/HILP and PG to the potential anticancer effects of the PG-CDs/HILP combination was investigated using cell viability, confocal imaging, apoptosis, and cell migration studies in addition to molecular docking.

## 2 Materials and methods

### 2.1 Materials

D-glucose (≥99.5%), petroleum ether (≥90%), sodium hydroxide, hydrochloric acid (36.5%–38%), ethanol (96%), methanol (≥99.8%) and dimethyl sulfoxide (DMSO, cell culture grade) were purchased from Sigma-Aldrich Co., United States. Fetal bovine serum (FBS), Dulbecco’s modified Eagle medium (DMEM), and phosphate buffer saline (PBS) were purchased from Gibco or Lonza, De Man, Rogosa and Sharpe (MRS) agar and broth (HiMedia Laboratories, India). Proteinase K (Enzynomics, South Korea, Catalog number: EO0491, PCR grade) was purchased from Serva, United States. *L. plantarum* (LP) was isolated and identified using 16S rRNA (accession number OQ144638). *Serratia marcescens* (*S. marcescens*), obtained from the culture collection of the Microbial Biotechnology Lab, Institute of Graduate Studies and Research, Alexandria University, was used as a source for prodigiosin pigment production. The strain was previously identified as *S. marcescens* using VITEK 2 biochemical identification (Joyanes et al., 2001) at the Faculty of Medicine, Department of Microbiology, Alexandria University. Both bacterial strains were maintained as a glycerol stock at −80°C according to its appropriate growth medium. The human lung cancer A549 and colon cancer Caco-2 cell lines were obtained from the Center of Excellence for Regenerative Research in Medicine and its Applications (CERRMA), Faculty of Medicine, Alexandria University.

### 2.2 Methods

#### 2.2.1 Heat-inactivated *Lactiplantibacillus plantarum* (HILP) preparation and characterization

*L. plantarum* (LP) was isolated earlier from yogurt and identified using 16S rRNA (unpublished data). The 16S rRNA sequence of the isolate was deposited in GenBank under the accession number OQ144638. LP was grown on MRS agar, and cultured twice before being used in MRS broth (Lab M) for 24 h at 37°C. Stock cultures were kept in MRS broth supplemented with 30% (v/v) glycerol and then stored at −80°C. A single colony of an overnight LP culture was used to inoculate 100 mL of MRS broth in a 250 mL conical flask and the mixture incubated at 37°C for 24 h. Bacterial pellets were collected by centrifugation at 672 × *g* for 10 min and washed twice with PBS pH 7.2. A LP suspension adjusted to 28 * 10^12^ CFU/mL (equivalent to 8 mg/mL) in PBS pH 7.2 was heat-inactivated by incubation in a water bath at 75°C for 60 min (Sashihara et al., 2006). HILP cells were checked for viability by inoculating 100 μL of the HILP suspension on MRS agar plates followed by incubation at 37°C for 72 h.

The quality of HILP was assessed by transmission electron microscopy (TEM 100 CX, Jeol-Japan) and laser scanning confocal microscopy (LCSM, Leica TCS SPE using the Leica LAS X interface). For TEM, samples were rapidly immersed in glutaraldehyde and fixed for at least 1 h at ambient temperature (∼23°C). This was followed by fixing the cells in osmium tetroxide and including them in agar. The specimens were dehydrated in ethanol and propylene oxide at decreasing concentrations before being implanted in Spurr’s plastic. Semi-thin pieces were cut from the blocks with a glass knife, and blocks were selected for thinning. Thin pieces were cut using diamond knives on copper grids and impregnated with uranyl acetate and lead citrate (Graham and Orenstein, 2007). For LCSM, HILP cells were fixed in 10% neutral buffered formalin for 10 min, washed twice with PBS pH 7.4, suspended in PBS, stained with the nucleat stain Hoechst, and finally mounted.

#### 2.2.2 Carbon dots preparation and characterization

Carbon dots (CDs) were prepared from D-glucose using a reported method (Seven et al., 2021) with slight modification. D-glucose (30 g) was dissolved in 200 mL distilled water (DW) and the solution was heated up to 200°C for 30 min and kept at 200°C for 5 h in a Teflon-coated autoclave reactor. The mixture was left to cool down at room temperature and centrifuged for 20 min at 13,221 × *g* product. The supernatant was filtered through 0.2 µm bacterial filter and adjusted to neutral pH using super saturated NaOH solution. The sample was dialyzed against DW using a dialysis membrane (VISKING^®^ dialysis tubing MWCO 1 KDa from Oxoid) for 3 days, changing water every 12 h. The dialyzed solution was lyophilized to yield a powder product.

CDs were characterized for morphology by TEM by examining a dried CDs dispersion placed onto a copper-coated TEM grid. The zeta potential (ZP) of CDs was measured using Zetasizer^®^ Nano ZS series DTS 1060, Malvern Instruments S.A, Worcestershire, United Kingdom. The optical properties of the prepared CDs were investigated by UV-vis absorption spectroscopy (Thermo Scientific-VISION pro- Software V4.30) and by recording their photoluminescence (PL) spectra at pH 5 and pH 7 (PerkinElmer LS55). The quantum yield (QY) of CDs was determined using quinine sulphate as standard as reported (Lawson-Wood et al., 2018). For Fourier-transform Infrared (FTIR) spectroscopy (Spectrum TwoTM), CDs were mixed with finely milled KBr (1:100) and scanned in the frequency range 4,000–450 cm^−1^ (Ren et al., 2019). In addition, the X-ray diffraction pattern (XRD) of CDs was obtained using X-ray diffractometer (Bruker D2-Phaser; Madison, WI, United States) at room temperature. The operating parameters were 30 kV and 10 mA with Cu Kα monochromatic radiation at a wavelength of 1.54184 Å. Measurements were performed with diffraction angle (2θ) scanned in the range of 1°–100°, step size of 0.01° and a step time of 0.1 s.

#### 2.2.3 Carbon dots functionalized HILP

A CDs-functionalized HILP hybrid (CDs/HILP) was prepared by loading CDs into HILP according to a reported protocol (Isabella et al., 2018). In brief, 1 mL of HILP suspension in PBS pH 7.4 (8 mg/mL PBS) was incubated for 4 h at 30°C with an equal volume of CDs dispersion in PBS at a final concentration of 1 mg/mL with shaking at 200 rpm. CDs/HILP cells were collected by centrifugation at 24,192 × *g*, washed four times with PBS, and kept in PBS at 4°C.

To investigate the potential role of cell wall proteins in the uptake of CDs by HILP, these proteins were denatured by incubating HILP (8 mg/mL) with 100 μL of a proteinase enzyme (Proteinase K 20 mg/mL) according to the manufacturer’s instructions for 30 min at 56°C as the optimum temperature for the enzyme is 50–56°C. The treated HILP was then incubated with CDs for functionalization as described above.

The CDs/HILP hybrid was characterized for CDs entrapment by TEM and LSCM without staining. In addition, the CDs content of the hybrid was determined by centrifuging the CDs/HILP suspension and measuring the concentration of unentrapped CDs in the supernatant by UV-Vis spectrophotometry at λmax 280 nm. The entrapment efficiency (EE%) and loading efficiency (LE%) were calculated using the following equations:

$$Entrapment\;efficiency\;\%=\frac{Entraped\;CDs,mg}{Initial\;CDs,mg}\times 100$$

$$Loading\;efficiency\;\%=\frac{Entraped\;CDs,mg}{CDs/HILP,mg}\times 100$$

Finally, the stability of the CDs/HILP hybrid suspended in PBS pH 7.4 was examined by LSCM imaging of the hybrid at the time of preparation and following storage for 3 months at 4°C.

#### 2.2.4 Prodigiosin (PG) production and characterization

PG was produced by *S. marcescens*. An inoculum size of 5% was cultivated on a sterile 2% peanut medium (pH 7) and incubated in a shaking incubator at 180 rpm at 28°C for 48 h (Giri et al., 2004) and then PG was extracted as reported (Hubbard and Rimington, 1950). Briefly, 10% of NaOH was added and the culture medium was incubated for 2 h under light exclusion. An equal volume of absolute ethanol was added, and the medium shaken at 120 rpm for 2 h at room temperature. An equal volume of petroleum ether 60/80 was added to the culture medium and the mixture subjected to vigorous shaking in a separatory funnel for 5 min. After complete separation of the two layers, the aqueous layer was discarded, and the organic layer was collected in a beaker and the precipitated cell debris were removed by filtration through Whatman filter paper No 1. Petroleum ether was evaporated in a rotary evaporator at 40°C (Faraag et al., 2017) and the dried PG was stored at −20°C. Crude PG in methanol was purified using a C-18 silica gel column. Impurities were eluted by flushing with 2 volumes of ethyl acetate till fading of the characteristic red color. Purified PG was concentrated by rotary evaporation at 40°C. Purified PG was characterized by UV-Vis spectroscopy by scanning a methanolic solution in the range 400–800 nm and FTIR by scanning a PG-KBr disc in the frequency range 3,000–800 cm^−1^.

#### 2.2.5 PG-loaded CDs/HILP

The developed carbon dots/heat-inactivated *L. plantarum* (CDs/HILP) hybrid was employed as biocarrier for the delivery of PG using pristine HILP for comparison. Loading of PG into CDs/HILP or pristine HILP was performed according to a reported method (Youssof et al., 2019) with modification. Briefly, HILP or CDs/HILP cells were incubated with 1 mL of PG solution (0.1, 0.3, 0.5, 1, 2, and 5 mg/mL in methanol) for 2 h at 28°C in a shaking incubator at 200 rpm. PG-HILP and PG-CDs/HILP were separated by centrifugation at 24,192 × *g* for 5 min, washed twice with PBS pH 7.4, stored at 4°C protected from light for further use.

PG-HILP and PG-CDs/HILP were examined for PG entrapment by LSCM without staining and TEM. Moreover, PG content was determined by extraction of PG from the bacterial carrier by vigorous vortex in absolute methanol for 10 min. The concentration of extracted PG was determined by UV-Vis spectrophotometry at λmax 530 nm. The PG EE% and LE% were calculated as for CDs.

#### 2.2.6 *In vitro* PG release studies

The release of PG from PG-CDs/HILP in comparison with PG-HILP was determined at 37°C in PBS pH 5 and pH 7.4 using a dialysis method with shaking at 60 rpm (Danyuo et al., 2019). A 1 mL-aliquot of the PG-CDs/HILP or PG-HILP samples was introduced into a dialysis bag (VISKING^®^ dialysis tubing MWCO 12000–14000) which was placed in 10 mL of the release medium. The concentration of PG released at predetermined time intervals was measured by UV-Vis spectrophotometry at λmax 530 nm. The sample of release medium removed for analysis was replaced with an equal volume of fresh medium adjusted at 37°C. Additionally, the presence of PG in HILP-PG and HILP/CDs-PG samples was verified by LSCM microscopy before and after the release process.

#### 2.2.7 *In vitro* anti-cancer activity studies

##### 2.2.7.1 Cell viability by the MTT assay

For cell viability examination, Caco-2 and A549 cells were seeded at a cell density of 7,000 cells/well in 96-well plate and were cultured in DMEM supplemented with 10% FBS and 1% penicillin/streptomycin, maintained in an incubator with 5% CO_2_, and humidified air at 37°C. After overnight culture, the medium was aspirated and replaced with DMEM containing various concentrations of PG (0.25–50 μg/mL) (Prabhu et al., 2016), HILP, and CDs/HILP in the concentrations 13.3–267.4 μg/mL or PG combination systems containing the same PG concentrations.

PG was dissolved in absolute methanol while HILP cells were suspended in 1 mL PBS, and all diluted in DMEM to reach the specified concentration. DMEM containing methanol was used as vehicle control. Cytotoxicity was measured after 24 h. Cells were incubated with 10 µL MTT (5 mg/mL) for 4 h, and the formed formazan crystals were dissolved in 100 µL of DMSO and the absorbance was measured at 590 nm using Tecan microplate reader, United States. All results were presented as the means of 5 replica. The median inhibitory concentrations (IC50) values were calculated according to the following equation (Chou, 2010):

$$\%\;Cell\;viability=\frac{Absorbance\;of\;treated\;cells}{Absorbance\;of\;control\;cells}\times 100$$

##### 2.2.7.2 Cellular uptake and distribution by LSCM

Caco-2 cells were cultured in the logarithmic growth phase in 6-well plates. Cells (300 000 cells/well) were treated with 20 μg/mL of the test preparations for 24 h. Subsequently, cells were stained with 4′,6-diamidino-2-phenylindole (DAPI) for nuclear staining (Wang et al., 2017) and imaged by LSCM.

##### 2.2.7.3 Analysis of the apoptotic effect of PG-CDs/HILP

The apoptotic effect of the test preparations was assessed using Caco-2 cells. These were seeded in 6- well plates at density of 3 × 10^5^ cells/well and treated with 20 μg/mL PG, PG-CDs/HILP, PG/HILP, and HILP for 24 h. Apoptosis was quantified using annexin V-FITC/propidium iodide (PI) kit. Briefly, control and treated tumour cells were trypsinized and collected via centrifugation and washed once with PBS. Cells were subsequently stained with 5 µL annexin V-FITC and 10 µL PI for 15 min at room temperature and the percentage of apoptotic cells was determined by flow cytometry (Wang et al., 2017).

##### 2.2.7.4 Effect of PG-CDs/HILP on Caco-2 cell migration

The cell migration assay was performed by creating a scratch (wound) in a monolayer of Caco-2 cells using a micropipette tip at base line (0 h). The detached cells were removed by washing with PBS. Fresh medium (2 mL) with or without test samples was added afterwards and incubated for 24 h. The ability of cells to migrate and close the wound was monitored by microscopic imaging. Images were analyzed using ImageJ and the percentage wound closure was calculated using the following equation (Kramer et al., 2013):

$$\%\;Wound\;closure=\frac{Wound\;size\;at\;zero\;time-Wound\;size\;at\;24\;h}{Wound\;size\;at\;zero\;time}\times 100$$

##### 2.2.7.5 Molecular docking

The interaction between PG, the anticancer agent used in the study, and human cancer cells was investigated using a molecular docking approach. The PG structure used for the bioinformatic analysis was retrieved from ChEMBL database (https://www.ebi.ac.uk/chembl/) and from ZINC database (ZINC28570356). Using the retrieved Simplified Molecular Input Line Entry System (SMILES) layout for PG (CCCCCC1=C/C(=C/c2[nH]c(-c3ccc[nH]3)cc2OC)N=C1C), PG was screened using the Swiss Target Prediction tool (http://swisstargetprediction.ch/) to predict potential cellular targets in human cells. Analysis of the targets was performed to identify major pathways affected by PG using KEGG, a database resource for understanding high-level functions and utilities of the biological system. Predicted targets were sorted by scores and targets known to play a role in cell growth and proliferation (5–10 targets) were selected to be further used for docking using SWISSDOCK (http://www.swissdock.ch/docking). The 3D structures of the selected targets were downloaded from the protein databank (PDB) website (https://www.rcsb.org/).

#### 2.2.8 Statistical analysis

Statistical analysis was performed using Excel and GraphPad Prism 8 Software (GraphPad Software, Inc.). When more than two groups were compared, differences among the groups were determined through one-way (Analysis of variance) ANOVA with a Tukey *post hoc* test for unpaired non-parametric variables.

## 3 Results and discussion

Although probiotics are classically defined as live microorganisms that benefit the host, recent evidence indicated that viability of probiotics is not an essential requirement for promoting health benefits (Piqué et al., 2019). Dead probiotics referred to as inactivated probiotics or paraprobiotics provide several advantages in terms of safety, particularly to vulnerable populations such as immunocompromised patients (Nakai et al., 2021). They also display strain-dependent efficacy in several clinical conditions such as promoting cutaneous wound healing (Tsai et al., 2021), alleviating stress-related type 2 diabetes (Nam et al., 2022) and exerting anticancer activity via potent growth inhibitory and apoptotic effects on cancer cells (Hwang et al., 2022).

### 3.1 Heat-inactivated *Lactiplantibacillus plantarum* (HILP)

**FIGURE 1:**
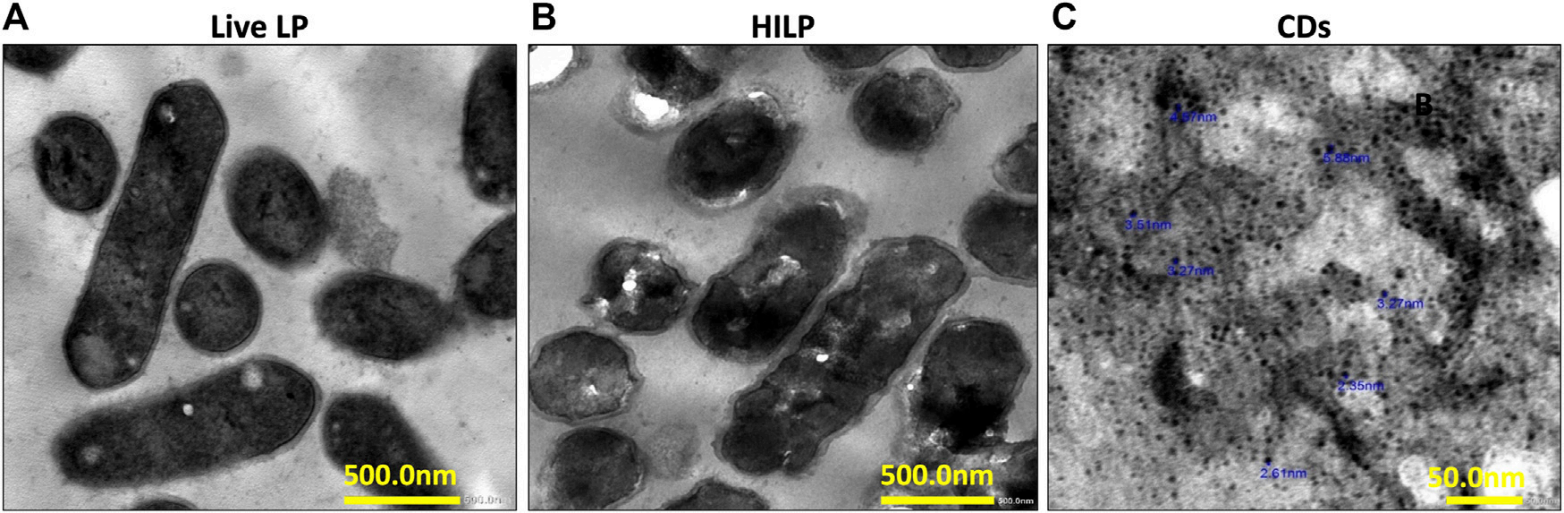
Representative transmission electron microscopy (TEM) images of **(A)** live *L. plantarum* (LP) cells; **(B)** heat-inactivated *L. plantarum* (HILP) cells showing maintained cellular morphology and intact cell wall (scale bar for both A and B represents 500 nm) and **(C)** D-glucose carbon dots prepared by hydrothermal bottom-up method (scale bar represents 50 nm).

Inactivated probiotics or paraprobiotics can be obtained using different methods, mainly sonication, irradiation, heat, and high pressure (de Almada et al., 2016). Thermal treatment was selected as inactivation method in the current study to conserve the surface structures of the bacteria while disabling its internal metabolism and retarding its ability to replicate (Rabiei et al., 2019). Bacterial inactivation was verified by lack of growth of HILP on MRS plates at 37°C for 72 h. TEM imaging of live LP and HILP (Figures 1A, B, respectively) showed that HILP cells retained their rod shape and intracellular contents as well as an intact cohesive cell wall.

### 3.2 Carbon dots (CDs)

In the preparation of CDs, the autoclave reactor/oven system kept the reaction solution at a constant temperature of 200°C for a fixed treatment time of 5 h. Constancy of the preparation conditions improve the homogeneity of CDs (Yoshinaga et al., 2017). Exposure of the D-glucose solution to the high temperature and pressure of the reactor resulted in several simultaneous decomposition reactions with browning of the D-glucose solution. Unreacted glucose molecules may adsorb to the CDs surface conferring hydrophilic and hydrophobic surface functionalities which may affect their properties and cellular interactions (Seven et al., 2019). The crude mixture containing CDs was purified and freeze dried.

TEM microscopy (Figure 1C) indicated that CDs were quasi spherical nanoparticles (NPs) having a mean diameter of 2.35–5.88 nm. Uniformity of CDs size can be attributed to maintenance of a constant temperature throughout the hydrothermal procedure. The UV/Vis spectrum of a CDs dispersion in deionized water showed absorption maxima at 280 nm and 350 nm (Supplementary Figure S1A). The PL spectra of CDs obtained at 280 nm excitation wavelength at pH 5 and pH 7 (Supplementary Figure S1B) indicated a slightly higher PL intensity at pH 5 with maintenance of this intensity following 72 h storage at 4°C. The QYs of the CDs calculated using quinine sulfate as standard was 8.95%. The relatively low QY of CDs can be attributed to the lack of doping atoms.

The FTIR spectrum of CDs (Supplementary Figure S1C) showed a broad band at 3,450 cm^−1^ corresponding to OH stretching, a peak at 2,930 cm^−1^ attributed to CH stretching, a peak at 1,630 cm^−1^ for C=O, a peak at 1,560 cm^−1^ corresponding to OH bending, a peak at 1,390 cm^−1^ for C=C, and a peak at 1,030 cm^−1^ for C-O. The peak in the fingerprint region of the CDs spectrum is characterized by bands of low intensity at 585 cm^−1^ for bending CH (Kurdekar et al., 2016). The FTIR spectrum of CDs indicated incorporation of functional groups of the D-glucose precursor on their surfaces. The XRD pattern of the prepared CDs (Supplementary Figure S1D) revealed the presence of hexagonal and orthorhombic carbon crystal structures. The peaks at 2θ of 29.04 and 41.46 correspond to hexagonal carbon (JCPDS #74-2330) with a crystallite size of 1.75 nm (based on the 002 peak) (Sangam et al., 2018). The peaks at 2θ of 21 and 23.45 correspond to orthorhombic carbon (JCPDS # 75-1621) with average crystallite sizes of 23.5 nm (Shaikh et al., 2019). The XRD peaks, interplanar spacing (d-spacing), full width at half maximum (FWHM), crystallite size calculated using the Debye–Scherrer equation and the Miller indices of the planes analyzed (hkl) are listed in Supplementary Table S1. Moreover, a light brown aqueous dispersion of CDs emitted bright green fluorescence upon exposure to UV radiation at 365 nm (Supplementary Figure S1E). Results obtained agreed with those reported for D-glucose derived CDs (Seven et al., 2019; Seven et al., 2021).

### 3.3 CDs labeled heat-inactivated *L. plantarum (*CDs*/*HILP)

HILP were functionalized with CDs and the obtained hybrid (CDs/HILP) was characterized by TEM and LCSM imaging for morphology. In addition, data were provided for the mechanism of CDs uptake by HILP and its retention stability.

#### 3.3.1 Morphological examination

**FIGURE 2:**
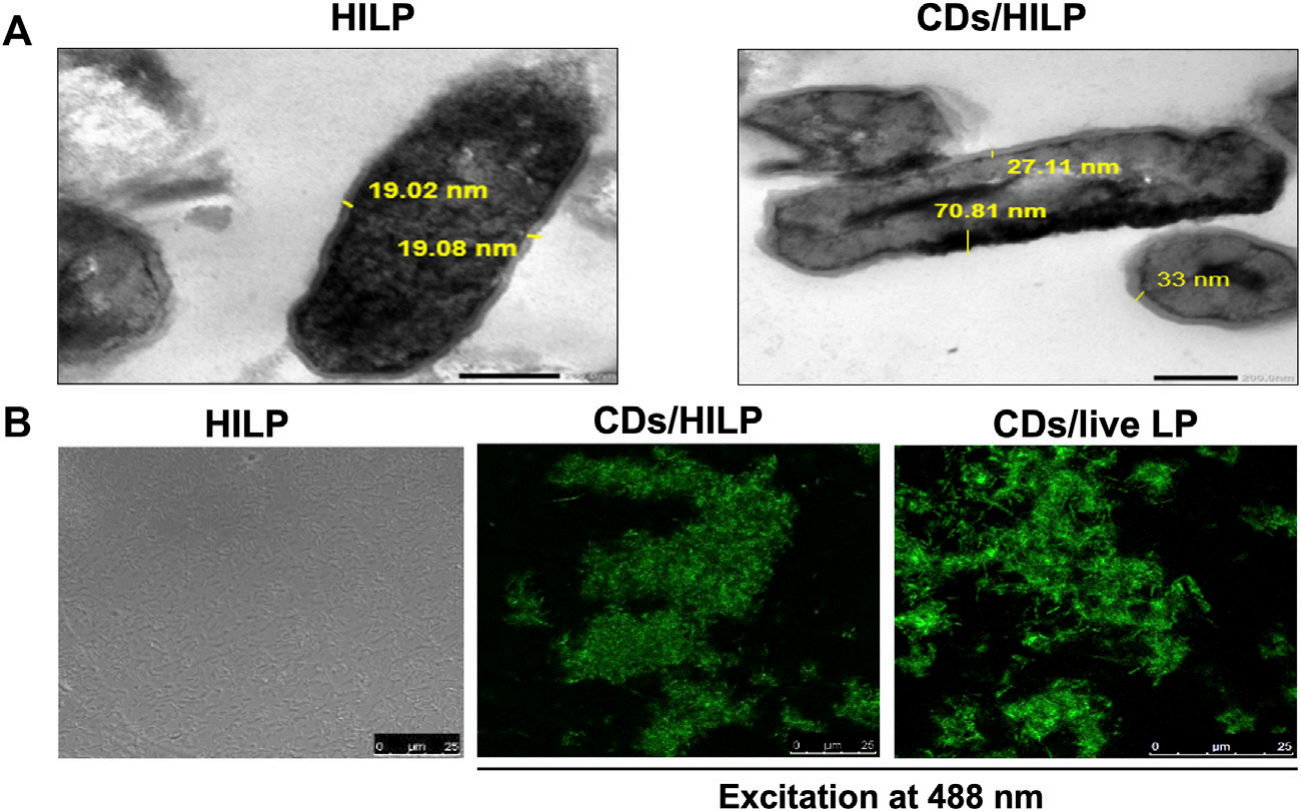
Microscopic examination of pristine heat-inactivated *L. plantarum* (HILP) and CDs-labeled HILP (CDs/HILP). **(A)** Transmission electron microscopy (TEM) images of pristine (HILP) at 30,000 × and CDs-labeled HILP (CDs/HILP) at 20,000 ×*.* **(B)** Laser scanning confocal microscopy images of pristine HILP (bright field) and CDs-labeled HILP (CDs/HILP) using CDs-labeled live LP (CDs/live LP) for comparison.

TEM imaging of pristine and CDs-labeled HILP (Figure 2A) revealed uniform distribution of dark spots of CDs in the cytoplasm of HILP, verifying cellular internalization. Moreover, the surface structure of the HILP cells was not damaged and the cell wall density and thickness of pristine HILP (⁓19 nm) were nonuniformly increased by CDs uptake (27–105 nm, Figure 2A). Preservation of cell wall integrity has vital implications in the physiology of Gram-positive bacteria as it conserves their integrity and shape which adds to the advantages of CDs as bioimaging agent for bacterial cells. Inorganic NPs such as zinc oxide, silver and silica NPs were shown to disturb the cell membrane of LP (Wang et al., 2021).

LSCM imaging (Figure 2B) illustrated changes induced by CDs to LP. While pristine HILP cells were non-fluorescent and non-aggregated, CDs labeling conferred intense green fluorescence to HILP implying efficient CDs uptake and induced aggregation of CDs/HILP cells. This cannot be attributed to changes in HILP surface properties as CDs-treated live LP cells also underwent aggregation. A similar phenomenon was observed with live *S. cerevisiae* yeast cells upon treatment with D-glucose derived CDs (Saleh et al., 2022). Aggregation was reported to occur among bacterial cells treated with carbon-based nanoparticles, such as single-walled carbon nanotubes, multi-walled carbon nanotubes, and graphene oxide nanoparticles (Dong et al., 2017). Aggregation of bacteria including *Lactobacillus* strains occurs for protection against environmental stresses (Zawistowska-Rojek et al., 2022). Physical stress mechanisms may trigger attractive forces of the hydrophobic and cell-protein interactions types between bacterial cells, leading to immediate aggregation (Burel et al., 2021). HILP aggregation entailed that heat treatment did not abolish the function of the surface protein layer necessary for aggregation (Saito et al., 2019). As bacteria aggregation promotes adherence to target cells, it may be beneficial to their biological and therapeutic activity. For instance, induced aggregation of lactic acid bacterial cells enhanced their adherence to Caco-2 intestinal epithelial cells *in vitro* (Okochi et al., 2017). However, CDs-induced aggregation appears to depend on the bacterial species and the CDs properties as the phenomenon has not been reported for other CDs-labeled bacteria (Nandi et al., 2015).

Results supported aptness of CDs as bioimaging nanomaterial for bacterial systems. Imparting fluorescence to microorganisms by CDs would facilitate their detection, analysis, quantitation and tracking for different identification, and theranostic purposes (León Anchustegui et al., 2021; Hallaji et al., 2022).

#### 3.3.2 CDs uptake and retention by heat-inactivated *Lactiplantibacillus plantarum* (HILP)

CDs uptake by HILP visualized by TEM and LCSM imaging (Figure 2) was verified by the relatively high EE (60%) and LE (6.9%) of CDs. This cannot be ascribed mainly to electrostatic interactions as both CDs (ZP -14 mV) and the bacterium bear a net negative charge. Although LP was reported to internalize the negatively charged SiO_2_ and Ag NPs (Wang et al., 2021), the high EE% of CDs by HILP cannot be explained solely by the rules of colloidal electrostatics (Westmeier et al., 2018). Alternative mechanisms might involve interaction of CDs with the peptidoglycan and glucose transporter proteins (GTPs) of the HILP cell membrane. The abundant hydrophilic groups on the peptidoglycan cell wall component of HILP may interact with the hydroxyl groups on the surface of CDs via hydrogen bonding (Fu et al., 2023). Moreover, uptake of CDs by LP may involve the interaction of CDs bearing intrinsically acquired surface glucose functional groups with the glucose transporter proteins (GTPs) of the LP cell membrane. GTPs are shared by most organisms for the absorption and metabolism of glucose at the cellular level (Thorens and Mueckler, 2010). To validate such an assumption, the uptake of CDs by pristine HILP as control and HILP treated with proteinase K before CDs labeling was examined by LSCM imaging. Proteinase K is a broad-spectrum, nonspecific, proteolytic enzyme widely used in molecular biology to degrade proteins. The enzyme remains active over a wide pH range (optimal between 6.5 and 9.5) and relatively high temperatures (optimal 50°C–65°C).

**FIGURE 3:**
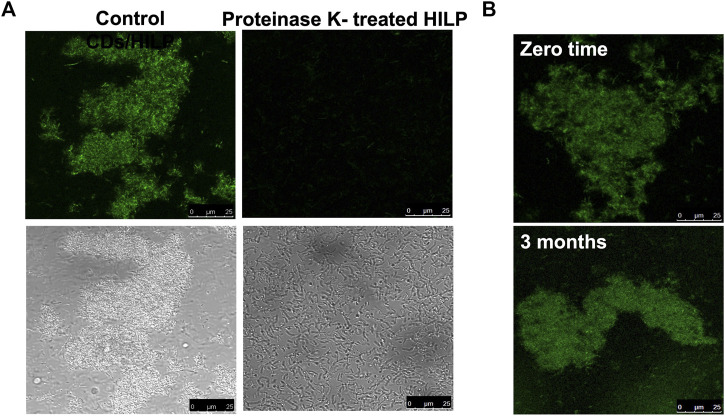
LCSM images for the uptake and retention stability of CDs by HILP. **(A)** Control HILP loaded with CDs showing aggregation and green fluorescence (Left panel). Treatment with Proteinase K for 30 min inhibited the development of green fluorescence and HILP aggregation (Right panel). **(B)** Retention of CDs by HILP upon incubation in PBS for 3 months at 4°C compared to freshly prepared CDs/HILP (zero time).

Figure 3A shows the effect of proteinase K treatment on the morphology of CDs/HILP. LSCM images revealed that the intense green fluorescence of CDs/HILP was not displayed by proteinase K treated HILP cells (Figure 3A, upper right panel), indicating inhibition of CDs uptake by HILP. The enzyme treatment also inhibited the aggregation of HILP as displayed by bright field imaging (Figure 3A, lower right panel). Accordingly, LSCM suggested preservation of cell membrane proteins following heat inactivation of LP and the implication of these proteins in the uptake of CDs by HILP cells as well as their aggregation. Findings corroborated those of a previous mechanistic study reporting accumulation of D-glucose CDs by a *S. cerevisiae* yeast strain having GTPs and failure of CDs uptake by another strain devoid of these membrane transporters (Seven et al., 2021).

Characterization of CDs/HILP for CDs retention stability upon incubation in PBS pH 7.4 for 3 months at 4°C indicated stability of the hybrid. The supernatant separated by centrifugation remained non-fluorescent upon UV exposure at 365 nm throughout the study duration. Moreover, LSCM examination at 488 nm excitation wavelength (Figure 3B) indicated that the intense green fluorescence of the freshly prepared CDs/HILP was retained throughout the study period. Stability of CDs/HILP is of great importance for tracking this hybrid structure both *in vitro* and *in vivo*. CDs are currently well established as cell imaging agents owing to their tunable fluorescence emission, brightness, and low toxicity (Hallaji et al., 2022). Findings of the present study support convenience of CDs as a simple, effective and economic alternative to fluorescent dyes and metal-based quantum dots (Hu et al., 2016; Xing et al., 2018; Zhao et al., 2022) as well as fluorescent proteins expressed by genetically engineered bacteria (Han et al., 2015; Stojanov et al., 2021) for bacterial identification and tracking.

### 3.4 Prodigiosin (PG)

The UV/Vis spectrum of purified PG obtained from *S. marcescens* (Agwa et al., 2018) showed a single sharp peak at 530 nm verifying purity of the sample (Supplementary Figure S2A). The FTIR spectrum of PG (Supplementary Figure S2B) showed a broad band at 3,413 cm^−1^ corresponding to NH absorption and bands at 2,968–2,845 cm^−1^ attributed to aromatic CH. The fingerprint region of the PG spectrum was characterized by bands of medium intensity: λmax 1,643 cm^−1^ (C = C), 1,458 cm^−1^ (bending CH) and 1,032 cm^−1^ (C–O). The PG spectrum obtained was similar to that reported previously (Alijani et al., 2022).

### 3.5 Prodigiosin-loaded CDs labeled heat-inactivated *L. plantarum*

#### 3.5.1 PG loading and retention by CDs/HILP

PG was also loaded into pristine HILP to examine the effect of CDs on PG loading. As reported in our earlier work involving PG loading into *Lactobacillus acidophilus* ghosts, the concentration of PG in the input solution was the main variable affecting PG loading (Saleh et al., 2022). In the present study, the effect of PG initial concentration on its loading into HILP and CDs/HILP was investigated while keeping all experimental variables constant (temperature 28°C, shaking time 2 h, and shaking speed 200 rpm). PG loading was determined by the recovery of entrapped PG by vigorous vortex of PG-HILP and PG-CDs/HILP with methanol, a good solvent for PG. Results listed in Supplementary Table S1 indicated that PG loading was enhanced by increasing the PG concentration in the input solution and reached a maximum at PG concentration 2 mg/mL under the study conditions. Moreover, PG recovery data were similar for HILP and CDs/HILP, implying lack of effect of CDs on PG uptake by the probiotic. An input PG solution 5 mg/mL was used in subsequent experiments. The calculated EE and LE of PG at the 5 mg/mL initial concentration were 14% and 4.66%, respectively.

To assess PG retention stability by HILP and CDs/HILP, PG-loaded probiotic suspensions were washed several times with PBS pH 7.4 and centrifuged for 5 min at 24,192 × *g* to remove unentrapped PG. Failure of the procedure to extract PG implied binding of PG to the HILP structures including the cell membrane and cytoplasm. PG was shown earlier to bind to *L. acidophilus* ghost membrane via the lipophilic domains of the probiotic cell membrane in addition to possible interaction of PG with plasma membrane components (Saleh et al., 2022).

#### 3.5.2 Characteristics of PG-CDs/HILP

**FIGURE 4:**
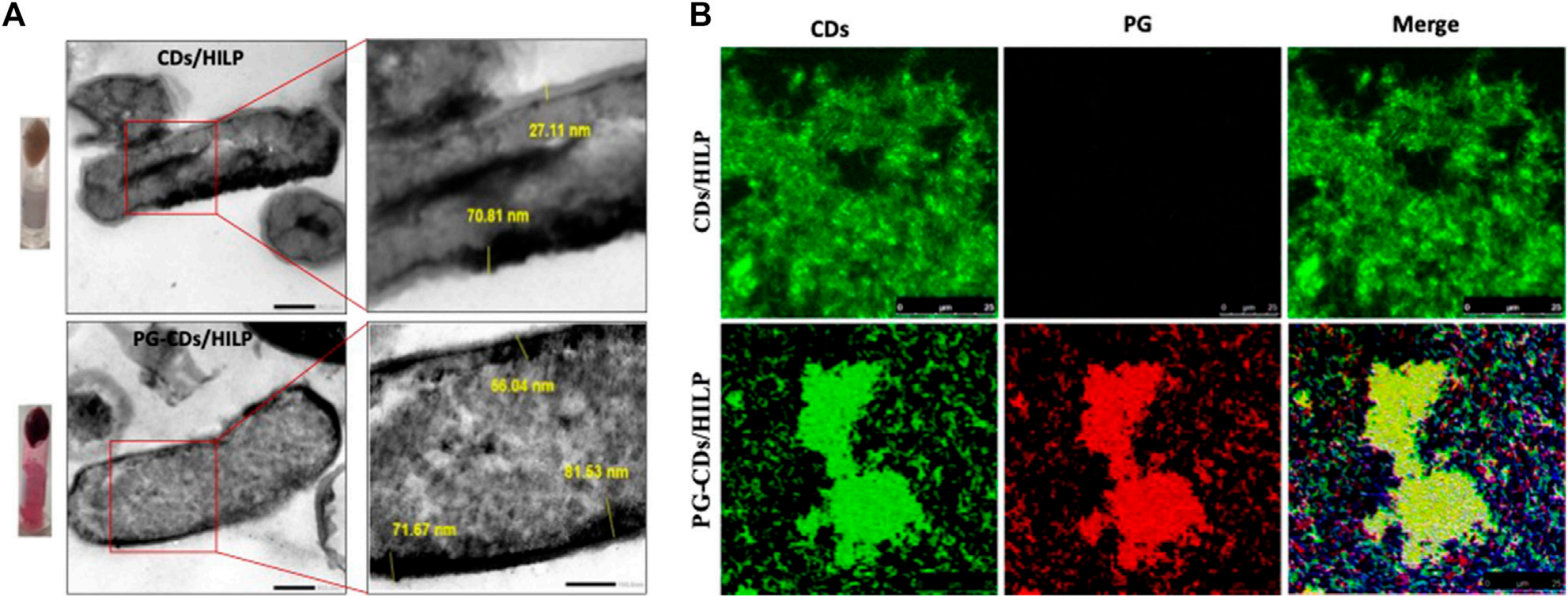
Microscopic examination of CDs/HILP and PG-CDs/HILP. **(A)** TEM images of CDs/HILP (Upper panel) and PG-CDs/HILP (Lower panel) at 25,000 × and their corresponding enlargements. Inverted Eppendorf tubes containing the pellets of CDs/HILP and PG-CDs/HILP are shown on the left. **(B)** LSCM images of green fluorescent CDs/HILP, red fluorescent PG-HILP and bifluorescent PG-CDs/HILP.

The developed PG-CDs/HILP combination was examined in comparison with the CDs/HILP biocarrier by TEM and LSCM (Figure 4). TEM imaging of CDs/HILP and PG-CDs/HILP (Figure 4A) and their corresponding enlargements indicated that PG loading did not affect the integrity of CDs/HILP cell walls. The cell wall density and thickness were rather increased, corroborating earlier findings using *L. acidophilus* ghost (Saleh et al., 2022). As shown in Figure 4B for the LSCM imaging of PG-CDs/HILP (lower panel) in comparison with CDs/HILP (upper panel), merging the green fluorescence of CDs/HILP and the red fluorescence of PG-CDs/HILP resulted in a bicolor fluorescent combination. Findings highlighted the bio-imaging properties of PG-CDs/HILP, of importance in the distinct tracking of CDs/HILP biocarrier and its drug cargo.

**FIGURE 5:**
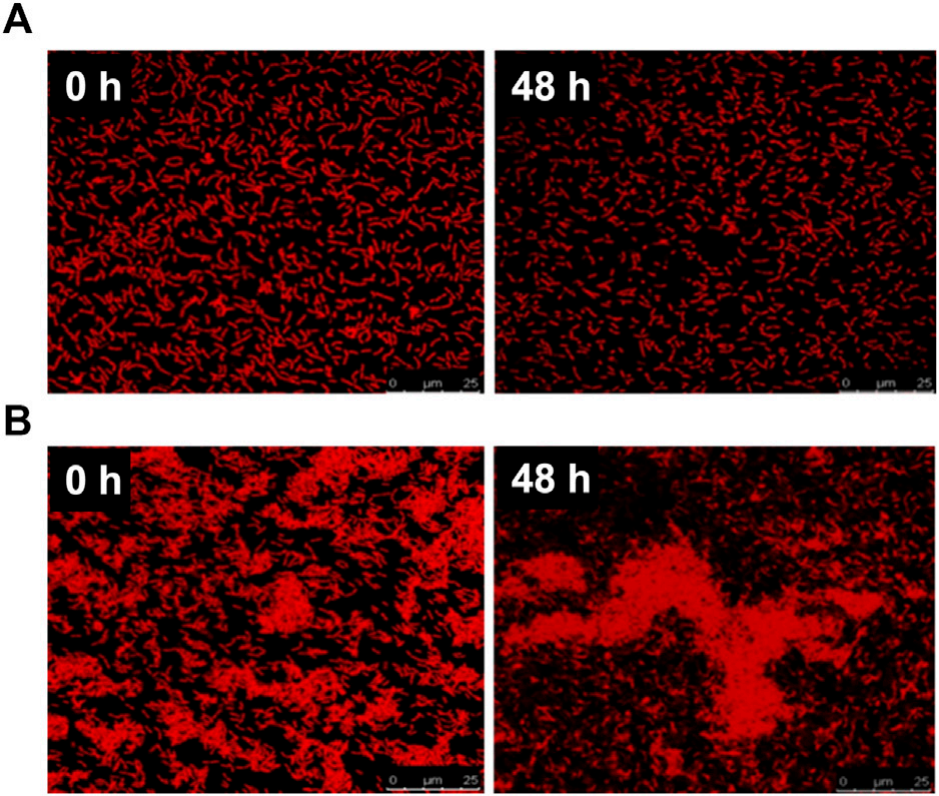
Release of PG from **(A)** PG-HILP and **(B)** PG-CDs/HILP after 48 h in PBS pH 7.4 at 37°C monitored by LSCM imaging.

The PG-CDs/HILP combination was further characterized for PG release in PBS pH 5 and pH 7.4 at 37°C and 100 rpm using PG-HILP for comparison. Sampling of the release medium at different time intervals revealed lack of PG release from both formulations for up to 48 h. Poor PG release was verified by extracting the pigment from HILP and CDs/HILP at the end of the release experiment in PBS pH 7.4 by vigorous vortex with absolute methanol. The amount of PG extracted was more than 96% of the initial amount loaded, confirming strong association of PG with HILP in both the absence and presence of CDs. Release data were further confirmed by LSCM imaging of PG-HILP (Figure 5A) and PG-CD/HILP (Figure 5B) at the beginning and the end of the release experiment. Results indicated retention of the PG red fluorescence by PG-HILP and PG-CD/HILP and confirmed strong aggregation of the PG formulation involving CDs/HILP as drug carrier.

Binding of PG can be explained at least in part by the affinity of the highly lipophilic PG molecules (log P_octanol-water_ 5.16) for the lipophilic domains of the bacterial cell wall in addition to its localization within the cytoplasm (Figure 5). Drug release properties from bacteria differ greatly from those of conventional biomaterial-based drug carriers which usually undergo marked physicochemical changes under the release conditions contributing to drug liberation. Although PG was released from chitosan microspheres (Dozie-Nwachukwu et al., 2017) and PLGA microparticles at pH 7.4 (Obayemi et al., 2016) due to the porosity and degradation of the polymer matrix, it was highly bound to *L. acidophilus* ghosts, forming a rather structurally stable entity that resisted simulated gastrointestinal fluids (Saleh et al., 2022). Strong PG binding to CDs/HILP probably generated an innovative bioactive probiotic combination as a potential anticancer biomedicine. This is presumably characterized by intrinsic biotargeting ability conferred by LP, bicolor fluorescence of the drug and the biocarrier, anticancer activity attributed mainly to PG and to a lesser extent LP and possibly involving CDs.

### 3.6 *In vitro* anticancer activity studies

These studies were performed on colorectal cancer (Caco-2) and human lung cancer (A549) cell lines to compare the activity of the probiotic hybrid (PG-CDs/HILP) relative to its components (PG and CDs/HILP as biocarrier) in addition to pristine HILP, PG-HILP and CDs. The effect of the test preparations on the viability of both cell lines was firstly assessed using the MTT assay. This was followed by elucidation of the possible mechanisms of anticancer activity of PG-CDs/HILP using Caco-2 cells. For all assessments, cells were seeded after performing the dye exclusion test using trypan blue (Strober, 2015).

#### 3.6.1 Cell viability

**FIGURE 6:**
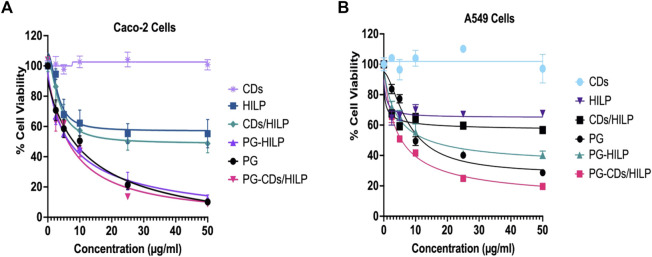
Cell Viability of **(A)** Caco-2 and **(B)** A549 cells using the MTT assay following 24 h treatment with the PG-CDs/HILP and its components CDs, PG, HILP, CDs/HILP and PG-HILP for comparison. Data points represent the mean ± SEM (*n* = 4). *p < 0.05 indicates a significant difference vs. the corresponding control group.

The cell viability data for the test preparations were obtained following a 24 h-cell treatment study as documented (Chen et al., 2021; Kim et al., 2022). A longer treatment time (48 h) resulted in reduced cytotoxicity. Cell viability profiles obtained (Figure 6) indicated a concentration-dependent cytotoxic effect within the concentration ranges of the test preparations except CDs which did not affect the viability of both cell lines in the 1–100 μg/mL concentration range. IC50 values are listed in Table 1. It could be generally observed that HILP was more cytotoxic to Caco-2 than A549 cells. PG and PG-containing formulations (PG-HILP and PG-CDs/HILP) were markedly more cytotoxic than those free of PG. Despite cytocompatibility when used alone, CDs considerably enhanced the cytotoxicity of PG-HILP (IC50 7.04 μg/mL vs. 12.7 μg/mL and 11.7 vs. 3,415 for Caco-2 and A549 cells, respectively). Finally, the PG-CDs/HILP hybrid exerted the highest activity against both Caco-2 and A549 cells (IC50 7.04 μg/mL and 11.7 μg/mL, respectively) and was thus, more active than its components, PG (IC50 7.29 μg/mL and 15.54 μg/mL) and CDs/HILP (29.6 μg/mL and 3,281 μg/mL) on Caco-2 and A549 cells, respectively.

**TABLE 1:** IC50 values for test preparations against Caco-2 and A549 cells following 24 h exposure.

Preparation	IC50, μg/mL	
	Caco-2 cells	A549 cells
CDs	—	—
HILP	36.7	1.07 × 10^7^
PG	7.29	15.54
CDs/HILP	29.6	3,281
PG-HILP	12.7	34.15
PG-CDs/HILP	7.04	11.7

Looking at the viability data for single agents, it was clear that CDs alone were well tolerated by both cell lines. The observed cytocompatibility of CDs (>95% cell viability) was consistent with reported findings (Jung et al., 2015; Tungare et al., 2020) but it is worth mentioning that the fabrication method of CDs affects its cytotoxicity. As CDs in this study were prepared from D-glucose, innate toxicity is rather unexpected. Unlike CDs, both HILP and PG affected the viability of cancer cells but to various extents as pristine HILP exerted low cytotoxicity against both cell lines whereas PG was expectedly much more cytotoxic. Remarkably, Caco-2 colorectal cancer cells were more sensitive to pristine HILP and PG compared to A549. The stronger effect of HILP on Caco-2 cells can be attributed to the intrinsic adherence of *L. plantarum* to adenocarcinoma cell lines such as Caco-2 cells and HT-29 (Zawistowska-Rojek et al., 2022). Other studies have also shown that the antiproliferative effect of *Lactobacilli* on cancer cells is cell line specific (Taherian-Esfahani et al., 2016). However, IC50 values of PG were slightly higher than the reported average value (2.1 μg/mL) in more than 60 different cancer cell lines (Islan et al., 2022). The complexity of the molecular mechanism of PG cytotoxicity which involves several biological processes necessary for cell viability might explain the observation. For instance, PG inhibits cell proliferation of colon cancer cells via disorganization of the F-actin structure (Guryanov et al., 2020), promotes apoptosis by inhibiting survivin (Hassankhani et al., 2015) and targets the expression of multiple apoptosis-related genes (Montaner and Pérez-Tomás, 2001; Saleh et al., 2022).

#### 3.6.2 Combination index and dose reduction index

Regarding the binary combinations CDs/HILP and PG-HILP, the cytotoxicity of the probiotic HILP was markedly increased against both cell lines, particularly A549, by embedding CDs into HILP and loading PG into HILP. The observed increase in HILP cytotoxicity induced by the cytocompatible CDs under the study conditions, could be explained by the CDs-induced aggregation of HILP as verified by LSCM in Figures 2B, 3. Induced aggregation of bacterial cells (*Lactobacillus rhamnosus*) was reported to enhance their adhesion to Caco-2 monolayer cells (Okochi et al., 2017). On the other hand, CDs may promote bacterial entry into the nucleus probably via nuclear import of CDs associated with the histone transport pathway (Jung et al., 2015). Enhanced cytotoxicity of HILP by PG loading is attributable mainly to the well-established anticancer activity of PG. Loading PG into the CDs/HILP hybrid biocarrier resulted in a combination with further enhanced cytotoxicity as indicated by IC50 values (Table 2). Anticancer activity enhancement appears to have involved the combined enhancing effects of CDs on HILP activity via aggregation-induced adhesion to cancer cells and enhanced cellular uptake of PG by CDs/HILP via endocytosis pathways.

**TABLE 2:** Combination index (CI) and Dose Reduction Index (DRI) of PG as anticancer agent and CDs/HILP hybrid as biocarrier either singly or in combination as PG-CDs/HILP on Caco-2 and A549 cell lines at Fa = 0.75 following 24 h exposure.

Cell type	CI	PG dose, µg	CDs/HILP dose, µg	PG DRI	CDs/HILP DRI
Caco-2	0.8555	20.578	205.684	1.29	12.9
A549	0.5286	54.163	234,949.00	1.89	8,208.3

**FIGURE 7:**
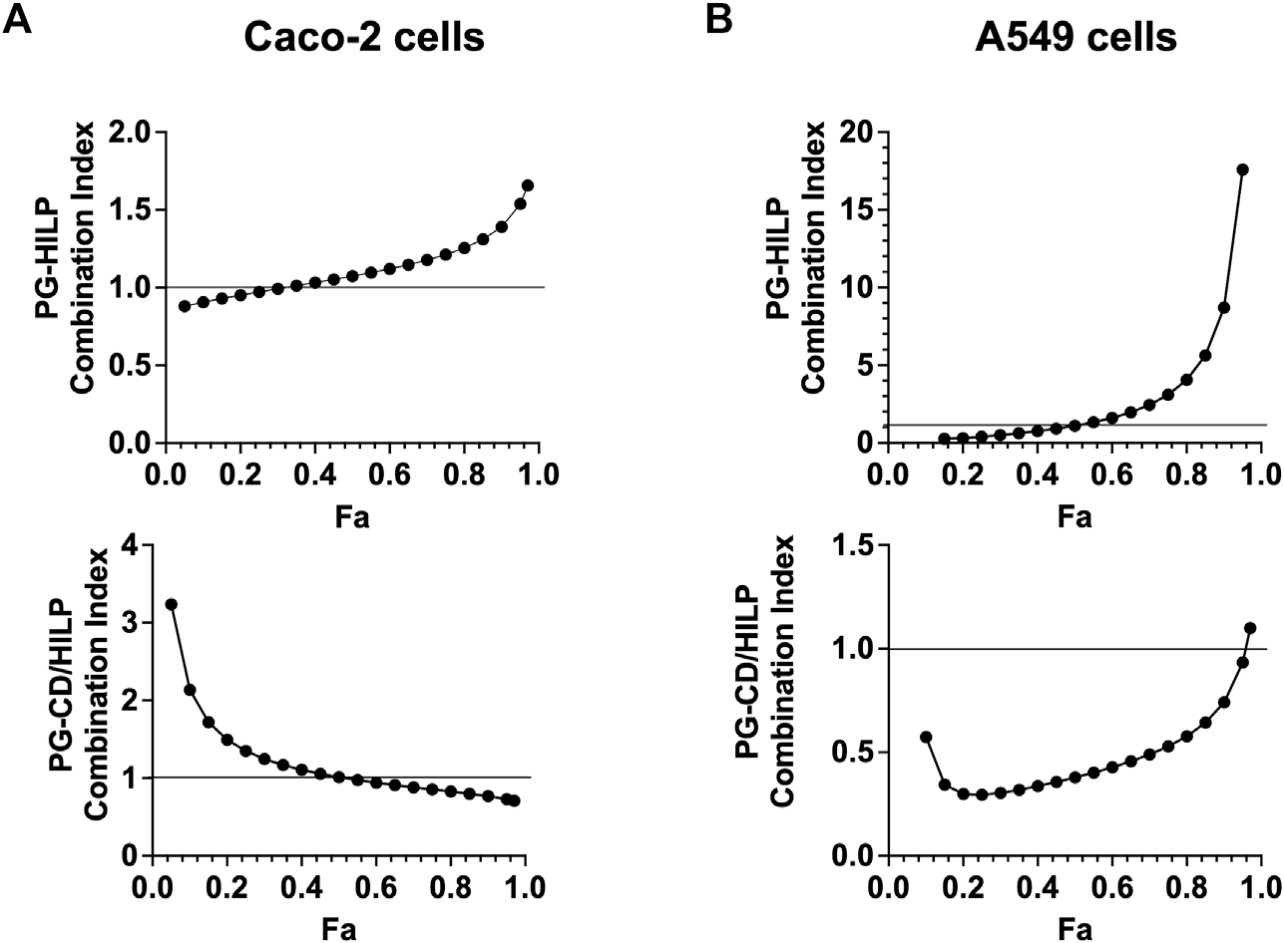
Combination index (CI) plot for PG-HILP and PG-CDs/HILP in **(A)** Caco-2 cells and **(B)** A549 cells (*n* = 4, Fa = fraction affected).

The Combination Index (CI) and the Dose Reduction Index (DRI) were computed according to Chou–Talalay method equation (Chou, 2010) to test for possible synergistic effects of the PG-CDs/HILP combination. CI implies synergistic, additive, or antagonistic effects of two bioactive agents in combination when CI < 1, CI = 1 or CI > 1, respectively. DRI indicates synergy of two bioactive agents and expresses the fold-decrease in the dose of either agent relative to their dose in the combination. It was interesting to note that in Caco-2 cells, the binary combination PG-HILP showed a synergistic effect at low inhibitory effects (Fa <0.5) whereas antagonistic effect was more obvious at higher levels (Fa >0.5). However, when functionalized with CDs (PG-CDs/HILP), the synergism was rather dominant at higher levels of inhibition (Fa >0.5) (Figure 7A), a desired effect in combinational anticancer therapy.

On the other hand, in A549 lung cancer cells (Figure 7B), the CI of PC-HILP was higher than 1 starting from Fa >0.5 which denoted antagonism. As PG alone was cytotoxic and A549 cells were less responsive to HILP than Caco-2 cells, it can be suggested that HILP attenuated the effect of PG. However, when CDs were added to the combination, synergism of PG-CDs/HILP was observed at almost all inhibition levels (Fa <0.9). Therefore, inclusion of CDs (Figure 7, lower panels) suppressed the antagonistic effects of the PG-HILP combination at Fa values higher than 0.5 on both Caco-2 and A549 cells and enhanced synergism at higher Fa for Caco-2 cells and at almost all Fa values (<0.9) for A549 cells. Enhancement of PG anticancer activity by the inclusion of CDs in the CDs/HILP biohybrid despite low cytotoxicity of CDs could be ascribed to a physical effect such as improved intracellular distribution of PG rather than a biological effect of CDs.

Data for CI and DRI at Fa = 0.75 are shown in Table 2. Synergistic effects of the PG-CDs/HILP combination (CI = 0.8555) against Caco-2 cells allowed for approximately 1.29-fold and 12.9-fold reduction in the dose of PG and CDs/HILP, respectively. Similarly, a potential 1.89-fold and 8208.3-fold reduction in the dose of PG and CDs/HILP, respectively could be speculated based on the synergistic cytotoxicity of the combination against A549 cells (CI 0.5286). Comparison of data for the two cell lines indicated that the DRI for PG was smaller for Caco-2 than A549 cells as PG is more cytotoxic to these cells (Table 2) while the DRI for CDs/HILP was greater for A549 cells as HILP has inherent greater tropism for colon cells. Such findings are of great importance regarding safety of the combination in biomedicine. Results also provided evidence for the benefit of combining PG with CDs/HILP in treating lung cancer given that some *Lactobacillus* species may act as effective adjuvants in the treatment of this cancer (Du et al., 2022).

### 3.7 Analysis of the anticancer activity of PG-CDs/HILP

The anticancer activity of PG-CDs/HILP and its components was examined using Caco-2 cells. Studies included cellular uptake and distribution of test formulations by LSCM, apoptotic activity of PG-CDs/HILP, its effect on Caco-2 cells migrating ability and finally identification of potential molecular targets of PG by molecular docking.

#### 3.7.1 Cellular uptake and distribution

**FIGURE 8:**
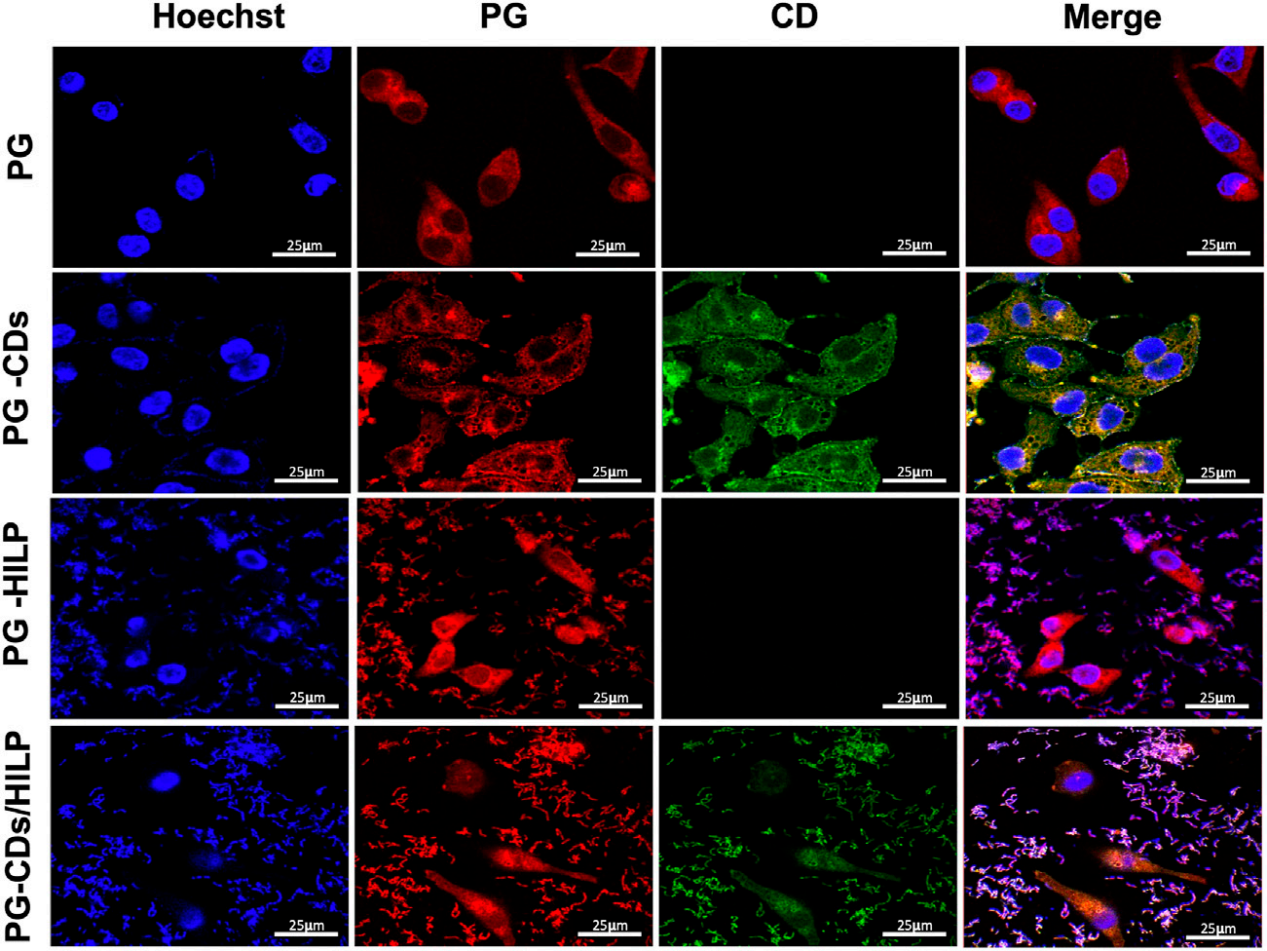
LSCM images of Caco-2 cells after treatment with prodigiosin (PG), PG loaded CDs (PG-CDs), PG-loaded into HILP (PG-HILP) and PG-CDs/HILP. The nuclei (blue) were labeled with DAPI. First row: red auto fluorescence of Caco-2 cells cytoplasm due to cellular uptake of PG; Second row: intracellular and nuclear delivery of both PG and CDs. Third row: PG delivery into the cytoplasm and nuclei of Caco-2 cells by HILP and Forth row: intranuclear delivery of both PG and CDs by HILP associated with nuclear changes.

As cell viability data suggested a potential role of CDs in the distribution and delivery of PG and HILP to cancer cells, cellular uptake by Caco-2 cells was further examined by LSCM. Possible intracellular delivery and distribution of PG-CDs/HILP and its components into Caco-2 cells was guided by the red and green fluorescence of PG and CDs, respectively (Figure 8). Treatment of Caco-2 cells with free PG induced changes in cell morphology associated with remarkable accumulation of PG in the cytoplasm (First row). This was not coupled with noticeable effects on nuclei. Examination of the effect of the formulation components on PG cellular uptake indicated that CDs were also delivered to the cytoplasm and partially to the nucleus of Caco-2 cells without apparently affecting PG delivery. CDs were reported to enter into the nuclei and subcellular organelles of cancer cells (Unnikrishnan et al., 2020). Intracellular and nuclear delivery of both PG and CDs was verified by LSCM images in the second row. However, the cytoplasm showed some changes which were reflected in the increased cytotoxicity. Loading PG into HILP increased its nuclear delivery (Figure 8, third row) while its loading into CDs/HILP resulted in nuclear changes associated with obvious cellular and nuclear uptake of both PG and CDs (Figure 8, fourth row). It has been reported that LP leads nuclear breakdown in human malignant melanoma A375 cells via cleavage of the poly (ADP-ribose) polymerase, a protein involved in DNA repair (Park et al., 2020) which might explain our observations. Owing to CDs labeling of HILP, it was possible to visualize the interaction of CDs/HILP with the Caco-2 cancer cells as well as the effect of HILP on nuclei, both possibly contributing to the enhancement of PG cytotoxicity.

#### 3.7.2 Analysis of the apoptotic effect of PG-CDs/HILP

**FIGURE 9:**
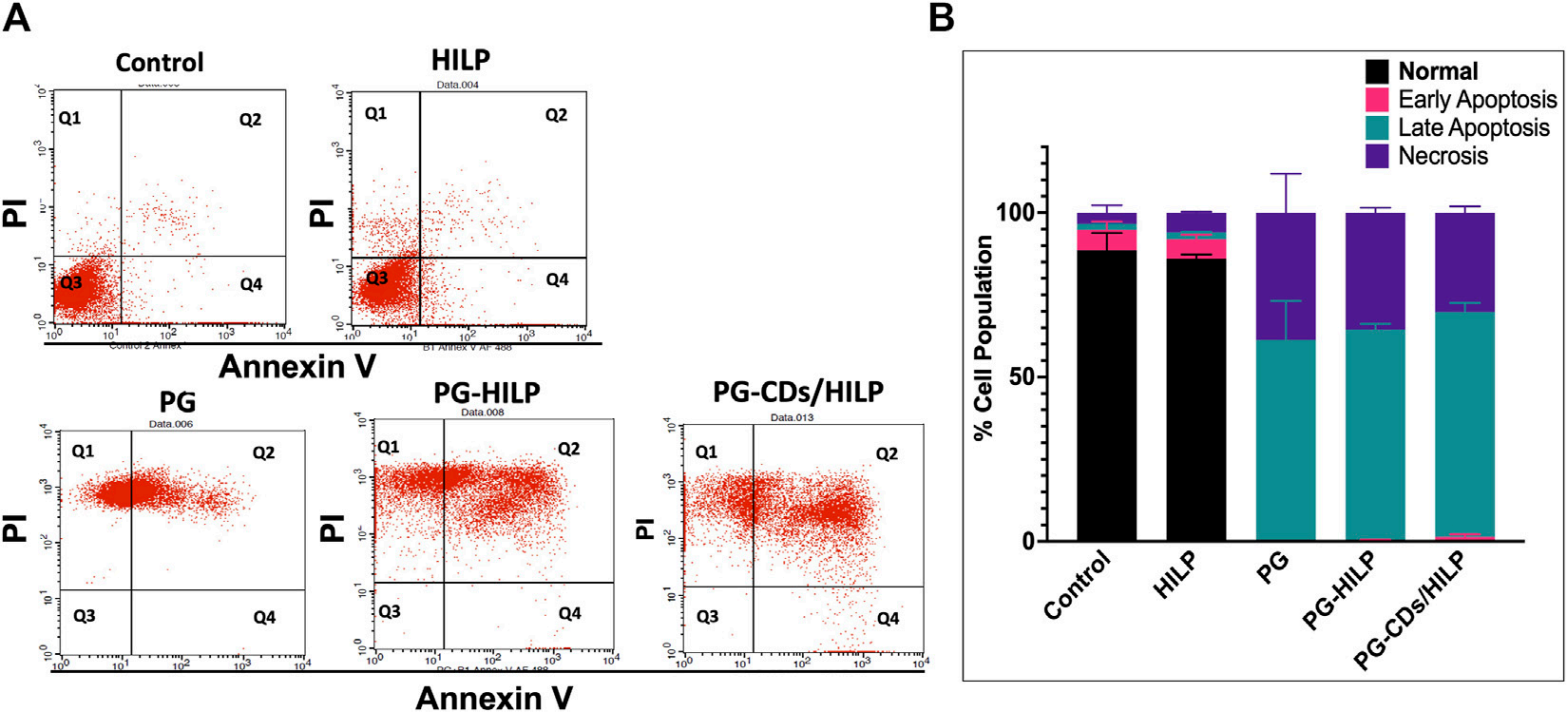
Apoptosis assay by Annexin V/Propidium iodide using flow cytometry on Caco-2 cells after treatment for 24 h with HILP and PG-containing formulations (PG solution, PG-HILP and PG-CDs/HILP) compared with vehicle-treated control. PG concentration was 20 μg/mL. **(A)** Dotplot showing (Q1) Necrotic cells, (Q2) late apoptotic cells, (Q3) living cells, and (Q4) early apoptotic cells. **(B)** Quantification of percentage cell population in each quadrant (*n* = 3).

To test for possible contribution of apoptosis to the cytotoxic effect of the PG-CDs/HILP hybrid to Caco-2 cells, the apoptotic effect of the hybrid and its individual components was assessed by flow cytometry. The number of apoptotic cells was measured by Annexin V/PI (propidium iodide) analysis following 24 h-treatment. As shown in Figures 9A, B, both untreated control and HILP-treated cells showed a relatively large percentage (>80%) of viable cells. Free PG treatment resulted in late apoptosis and an increase in the number of necrotic cells. Loading PG into HILP or CDs/HILP stimulated mainly late apoptosis and increased the number of necrotic cells. It has been reported that cytotoxicity of PG against different types of cancer cells involves a PG-induced increase in the acidity of lysozymes and stimulation of apoptosis (Narsing Rao et al., 2017). In the present study, PG-CDs/HILP treatment even increased the apoptotic stress on Caco-2 cells so that more cells entered late apoptosis compared to free PG. As pristine HILP exerted a minimal apoptotic effect, findings provided evidence for the interactive role of the CDs/HILP carrier with PG in the anticancer activity of the PG-CDs/HILP hybrid, verifying the synergism demonstrated by cell viability data.

#### 3.7.3 Effect of PG-CDs/HILP on Caco-32 cell migration

**FIGURE 10:**
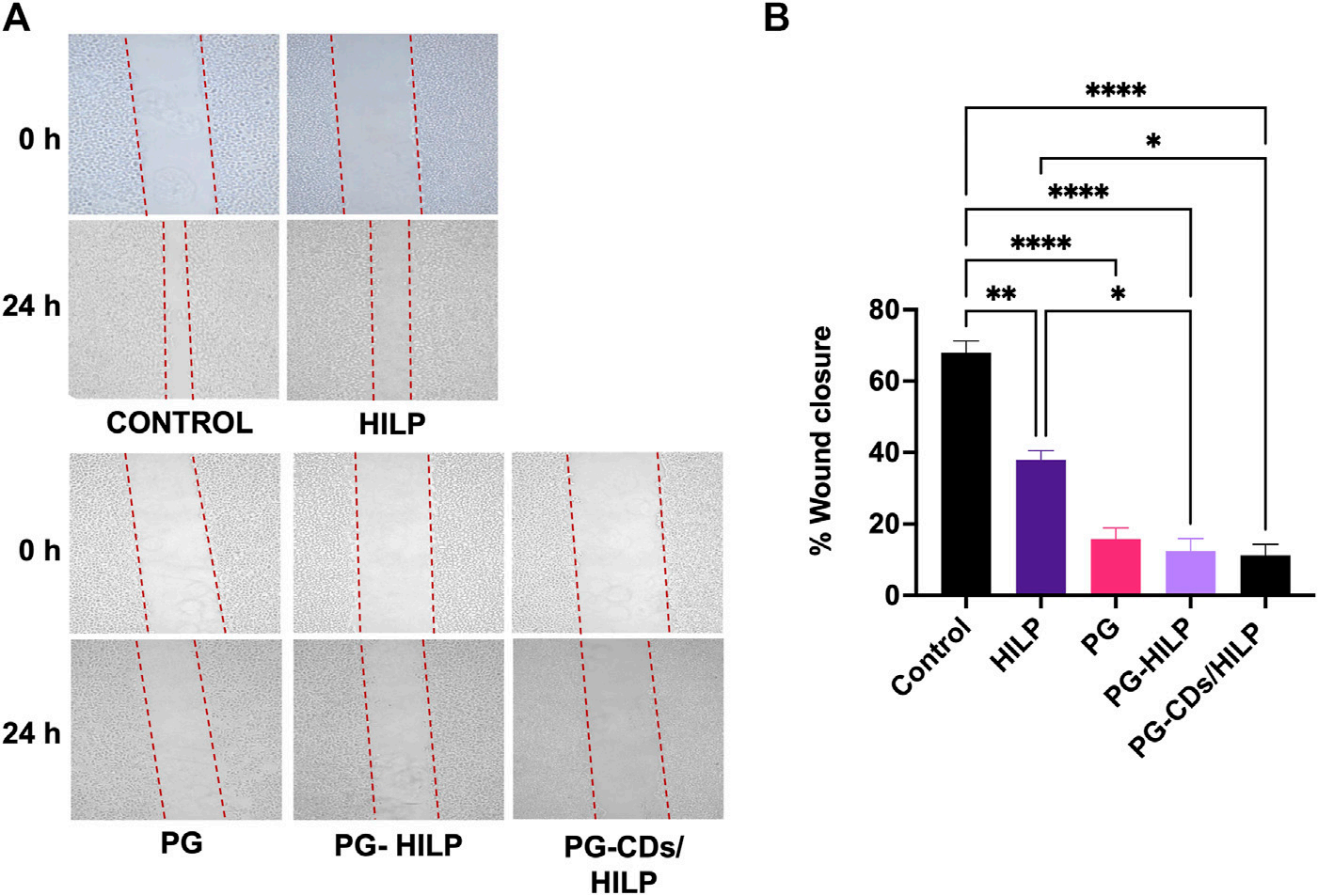
*In vitro* scratch (wound healing) assay for estimating the migration of Caco-2 cells following 24 h-treatment with HILP and PG-containing formulations (PG solution, PG-HILP and PG-CDs/HILP) compared with vehicle-treated control. PG concentration was 20 μg/mL **(A)** Representative microscopic images before and after treatment (×100 Magnification). **(B)** Quantification of wound closure each compared to 0 h (*n* = 3).

Results of the scratch assay (Figures 10A, B) demonstrated that migration of Caco-2 cells was significantly reduced by PG and the PG-containing preparations (PG-HILP and PG-CDs/HILP) compared with PG-free preparations. Moreover, inhibition of cell migration by PG-HILP and PG-CDs/HILP surpassed that of free PG, suggesting enhanced inhibition of invasion of colorectal cancer cells. Although PG-CDs/HILP achieved the greatest Caco-2 cell migration inhibition, the effect was not significantly different from that of free PG under the test conditions (24 h-treatment). Results implied that the significant inhibitory effect of PG on Caco-2 cell migration was not suppressed by the CDs/HILP biocarrier. Accordingly, the hybrid CDs/HILP offers advantages as a bioactive anticancer drug carrier by allowing bioimaging of the interaction of the drug delivery system with target cells and enhancement of its bioactivity.

#### 3.7.4 Prediction of prodigiosin molecular targets

**FIGURE 11:**
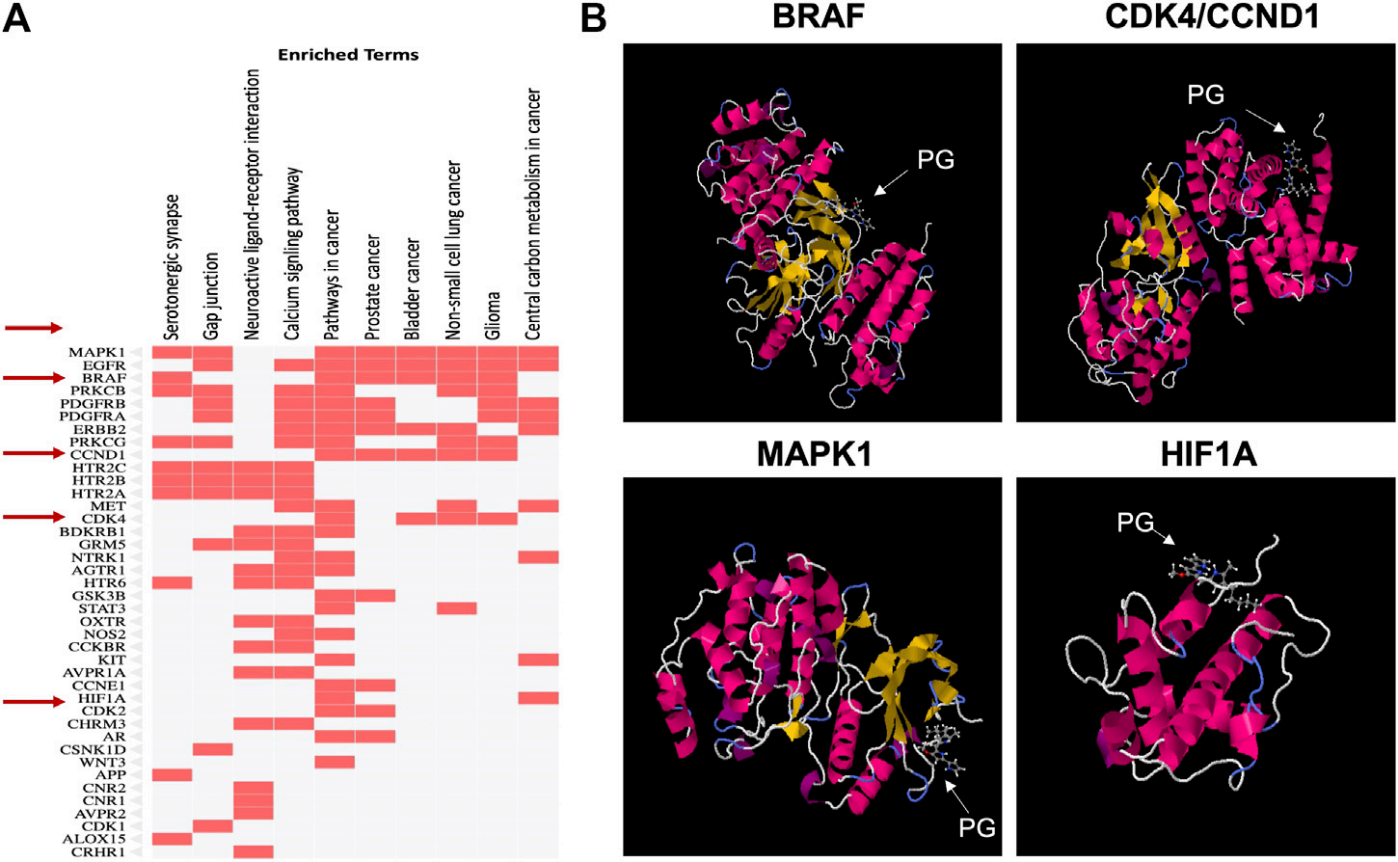
Prediction of prodigiosin molecular targets using the SWISSTargetPrediction tool. **(A)** Analysis of targets by KEGG pathway to identify those enriched for the selected BRAF, CCND1, MAPK1 and HIF1A proteins involved in cancer and **(B)** Docking for the four selected proteins.

For further elucidation of the molecular mechanism involved in PG anticancer activity, molecular targets of PG were predicted using SWISSTargetPrediction tool as shown in Figure 11. KEGG pathway analysis showed enrichment of the top 40 targets for pathways related to different cancers such as prostate cancer, bladder cancer, non-small cell lung cancer and glioma (Figure 11A). From the identified targets, docking was attempted for proteins known to play a role in cancer, like BRAF, CCND1, MAPK1 and HIF1a (Figure 11B). The pose of the prodigiosin ligand with the respective protein was selected based on affinity score and lowest energy (Table 3).

**TABLE 3:** Affinity score and estimated energy for the bonding of prodigiosin to the four selected docking proteins.

Protein	PBD ID	Full fitness (kCal/Mol)	Estimated ΔG (kCal/Mol)
BRAF	3Q4C	−30,003.12	−8.10
CCND1/CDK4	2W9Z	−2,877.71	−9.13
MAPK1	4G54	−1,662.80	−8.46
HIF1A	1L8C	−988.13	−8.63

Molecular docking revealed that PG possibly interacts with CCND1 and MAPK1 which are both responsible for growth promotion and the proliferation process. Several studies have identified these genes as core targets in colorectal cancer (Shi et al., 2021). Such an interaction would affect cellular proliferation as observed in the MTT assay results and explains the decreased cell viability induced by the PG preparations. Gene ontology for biological processes analysis showed enrichment for proliferation and apoptosis processes (data not shown) aligning the results obtained from apoptosis analysis.

HIF1a is a known key player in progressed and drug-resistant tumors as it responds to hypoxic stress to promote proliferation and angiogenesis and thus support cell migration and consequently metastasis. Upregulation of HIF1a is often correlated with poor prognosis and was involved in 5-fluorouracil resistance of colorectal cancer cells (Dong et al., 2022). Being a possible target for PG clarifies the reduced migration observed upon treatment with PG or PG-containing preparations. Targeting both proliferative and resistance markers render PG a potential therapeutic option, especially in drug-resistant colorectal cancer.

## 4 Conclusion

An innovative multifunctional fluorescent biocarrier consisting of a bioactive paraprobiotic (heat-inactivated *L. plantarum,* HILP) labeled with green fluorescent D-glucose carbon dots (CDs) for drug delivery applications was developed. Efficient uptake of CDs and preservation of their fluorescence by HILP in aqueous media verified aptness of CDs/HILP as a trackable drug carrier. Application of CDs/HILP as biocarrier for the microbial anticancer prodigiosin pigment resulted in a stable bioinspired anticancer structure with bicolor distinct red/green fluorescence emission. This contributed to a better understanding of the anticancer effects of both prodigiosin and CDs/HILP biocarrier on colorectal Caco-2 cancer cells. Accordingly, the developed CDs/paraprobiotic hybrid offers promise as a safe, trackable, and scalable drug biocarrier combining the safety, sustainability, bioactivity, and affinity of HILP for intestinal cells along with the bioimaging ability and cellular activity of CDs. Findings highlighted the possibility of applying pharmaceutical technologies adopted for the formulation of biomaterial-based drug delivery systems to enhancing the performance of probiotics as biocarriers. These involve mainly customization of probiotics and their different structures such as ghosts and inactivated cells to acquire bioimaging, therapeutic, preventive, and theranostic functionalities. On the other hand, probiotics as biocarriers may enrich the drug delivery field with safe, economic, and bioactive drug carriers with favourable physiological and customizable therapeutic activities. Utilization of bacteria as drug carriers may contribute to reshaping drug delivery and other biomedical bionanotechnological applications in the future.

## Data Availability

The original contributions presented in the study are included in the article/Supplementary Material, further inquiries can be directed to the corresponding author.
