# Supplementary material for: Carbon dots labeled Lactiplantibacillus plantarum: a fluorescent multifunctional biocarrier for anticancer drug delivery
Source: Front Bioeng Biotechnol. 2023 May 25;11:1166094. doi: 10.3389/fbioe.2023.1166094 (PMC10248154; doi:10.3389/fbioe.2023.1166094)
Supplement: Supplementary file 1 [file DataSheet1.pdf]

Supplementary material

Carbon dots labeled *Lactiplantibacillus plantarum:* A fluorescent multifunctional biocarrier for anticancer drug delivery

Noor A. Abdullah[1], Hoda E. Mahmoud[1], Nefertiti A. El-Nikhely[1], Ahmed A. Hussein[1] and Labiba K. El-Khordagui[2]

[1]Department of Biotechnology, Institute of Graduate Studies and Research
[2]Department of Pharmaceutics, Faculty of Pharmacy, Alexandria University

SUPPLEMENTARY TABLE 1

X-ray diffraction (XRD) parameters of the prepared D-glucose CDs

XRD Peaks	D-spacing (nm)	FWHM	Crystallite size (nm)	***hkl*** **(phase)**
21.00	0.423	0.36	23.45	011-**O**
23.45	0.379	0.36	23.55	002-**O**
29.04	0.307	4.87	1.76	002-**H**
41.46	0.217	-	-	101-**H**

SUPPLEMENTARY TABLE 2

Recovery of prodigiosin (PG) loaded into heat inactivated *L. plantarum* (HILP) and CDs/HILP

Initial PG concentration, mg/mL	PG recovered from HILP, µg/mL	PG recovered from CDs/ HILP, µg/mL
0.1	59	55
0.3	155	158
0.5	344	341
1	596	589
2	699	694
5	730	733

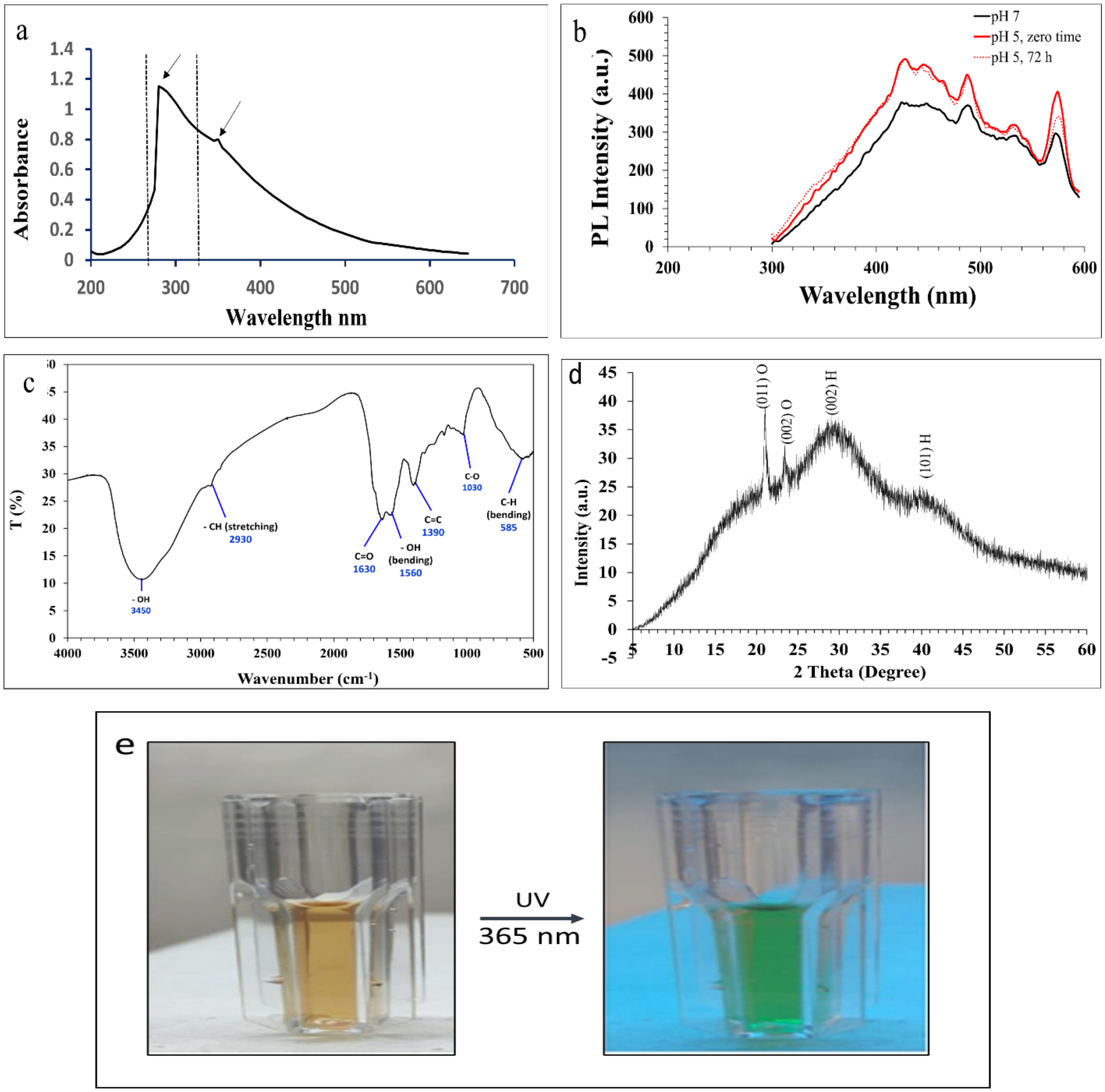

SUPPLEMENTARY FIGURE 1

Properties of the prepared carbon dots: a) UV/Vis spectrum of CDs dispersion in deionized water. The arrows point to the maximum absorbance around 280 and 350 nm; b) Photoluminescence (PL) spectra for CDs at 280 nm excitation wavelength at pH 7 and pH 5 at zero time and following storage for 72 h at 4°C; c) FTIR spectrum; d) XRD pattern and e) Digital images of CDs dispersion in deionized water before and after UV excitation at 365 nm.

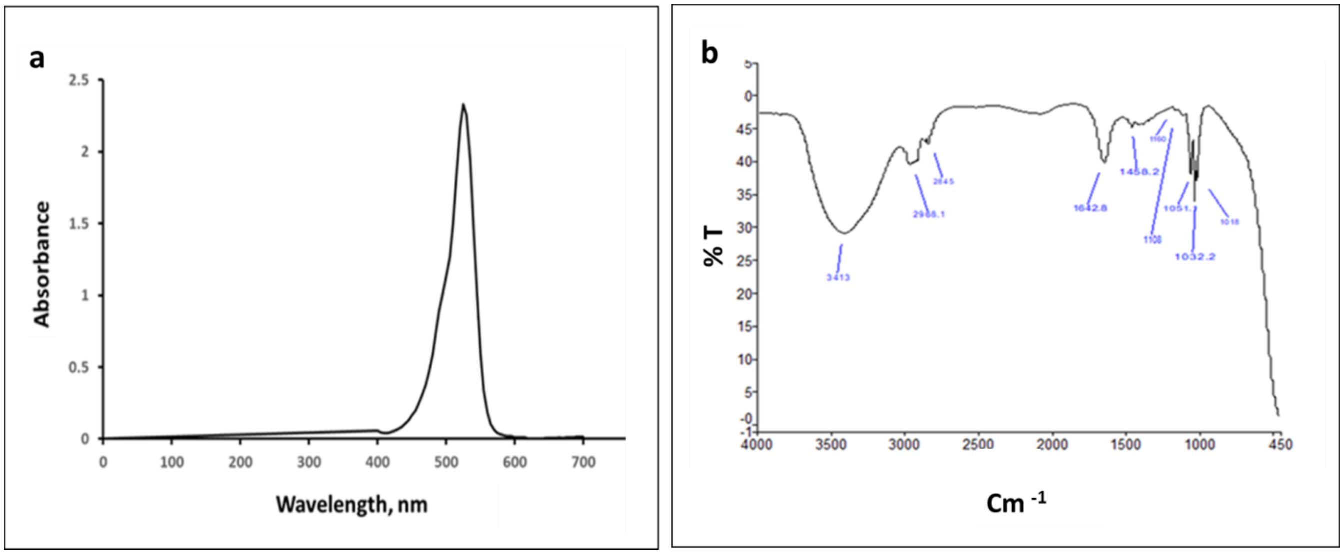

SUPPLEMENTARY FIGURE 2

Spectral properties of purified prodigiosin: a) UV-Vis spectrum and b) FTIR scan
